# Type 1 interferonopathy presenting as juvenile idiopathic arthritis with interstitial lung disease: report of a new phenotype

**DOI:** 10.1186/s12969-020-00425-w

**Published:** 2020-05-12

**Authors:** S. L. N. Clarke, L. Robertson, G. I. Rice, L. Seabra, T. N. Hilliard, Y. J. Crow, A. V. Ramanan

**Affiliations:** 1 Department of Paediatric Rheumatology, Bristol Royal Hospital for Children, Upper Maudlin Street, Bristol, BS2 8BJ UK; 2 MRC Integrative Epidemiology Unit & School of Population Health Sciences, University of Bristol, Bristol, UK; 3 Department of Rheumatology, Derriford Hospital, Plymouth, UK; 4 Division of Evolution and Genomic Sciences, School of Biological Sciences, Faculty of Biology, Medicine & Health, University of Manchester, Manchester Academic Health Science Centre, Manchester, UK; 5 Paris Descartes University, Sorbonne-Paris-Cité, Laboratory of Neurogenetics & Neuroinflammation Institut Imagine, Hôpital Necker Enfants Malades, Paris, France; 6 Department of Paediatric Respiratory Medicine, Bristol Royal Hospital for Children, Bristol, UK; 7 Centre for Genomic and Experimental Medicine, MRC Institute of Genetics & Molecular Medicine, University of Edinburgh, Edinburgh, UK; 8 School of Translation Health Sciences, University of Bristol, Bristol, UK

**Keywords:** SAVI, Autoinflammatory, Interstitial lung disease, Interferon, Juvenile rheumatoid arthritis

## Abstract

**Background:**

STING-associated vasculopathy with onset in infancy (SAVI) is a type 1 interferonopathy manifesting as a pulmonary and vascular syndrome resulting from gain-of-function mutations in *TMEM173*, the gene encoding STING. Familial reports in the literature are sparse.

**Case presentation:**

We report a case series of SAVI in a three generation kindred, with a phenotype of interstitial lung disease (ILD) and rheumatoid factor positive polyarticular juvenile idiopathic arthritis (JIA). Current and historical medical records were reviewed for clinical and laboratory information. Whole blood from cases 1 and 2, plus stored appendicectomy tissue from case 3, underwent DNA sequencing of the *TMEM173* gene. Peripheral blood RNA was obtained from cases 1 and 2 for functional assessment of the *TMEM173* mutation. DNA sequencing identified the same heterozygous *TMEM173* mutation (c.463G > A; p.Val155Met) in all three cases, consistent with a diagnosis of the autosomal dominant condition SAVI. Functional assessment of this mutation identified a prominent interferon signature which was confirmed on repeat testing.

**Conclusions:**

SAVI presented in this family as ILD with early onset juvenile rheumatoid arthritis. This condition should be considered in all rheumatoid arthritis patients with early-onset ILD and in all JIA patients with ILD.

## Background

First described in 2014 [1], STING-associated vasculopathy with onset in infancy (SAVI) is a type 1 interferonopathy manifesting as a pulmonary and vascular syndrome resulting from gain-of-function mutations in *TMEM173*, the gene encoding STING. To date there are only three reports of affected families in the literature [2–4].Here we describe a three generation kindred with a phenotype of interstitial lung disease (ILD) and rheumatoid factor positive (RF^+^) polyarticular juvenile idiopathic arthritis (JIA), segregating as a dominant trait due to a recurrently described V155M mutation in STING. This report extends the SAVI phenotype and emphasises the need to consider this diagnosis in patients with JIA or early onset rheumatoid arthritis plus ILD.

## Case presentation

The index case (case 1) is a 15-month-old Caucasian girl born by spontaneous vaginal delivery at 28 weeks of gestation, weighing 1056 g (30th centile). Her course on the neonatal unit was unremarkable given her gestation. She was discharged home at 3 months of age with presumed chronic lung disease of prematurity on 0.06 L low flow oxygen and no evidence of skin disease. She was readmitted 1 month later with failure to thrive and respiratory difficulties. She received treatment for a lower respiratory tract infection with antibiotics and high flow oxygen. She continued to deteriorate, requiring intubation and ventilation at 5 months of age. Chest computed tomography identified widespread ground glass interstitial changes consistent with ILD. Lung biopsy showed interstitial thickening with type 2 pneumocyte hyperplasia, but genetic testing for known pathogenic variants related to surfactant deficiency was negative. Results of her laboratory investigations are shown in Table 1.

**Table 1:** Clinical and laboratory findings of cases 1–3

	Case 1	Case 2	Case 3
Gender	Female	Female	Female
Age at presentation	Neonate	22 months	16 years
Failure to thrive	Yes	Unknown	Unknown
Rash	No	Yes	Unknown
ILD	Yes	Yes	Yes
Arthritis	No	Polyarticular JIA	Polyarticular JIA
CRP (mg/L)	56	< 5–46	3^a^
Hb (g/L)	7.1 (NR 11.4–14.1)	11.1 (NR 11.5–16.5)	12.1 (NR 11.5–16.5)
C3 (g/L)	1.47 (NR 0.9–1.8)	1.65 (NR 0.75–1.65)	Unknown
C4 (g/L)	0.24 (NR 0.1–0.4)	0.2 (NR 0.14–0.54)	Unknown
IgG (g/L)	11.49 (NR 2.4–8.8)	26.0 (NR 6.0–16.0)	10.10 (NR 6.0–16.0)
IgA (g/L)	0.66 (NR 0.1–0.5)	3.5 (NR 0.5–2.4)	3.06 (NR 0.8–2.8)
IgM (g/L)	1.36 (NR 0.2–1.0)	2.1 (NR 0.5–1.8)	1.26 (NR 0.5–1.9)
Anti-smooth muscle	Positive	Negative	Negative
ANA	Negative	1:320	1:80/negative^b^
ENA	Negative	Negative	Negative
Anti-dsDNA (mg/L)	Negative	Negative	1.2
RF (IU/mL)	Negative	123	797
Anti-CCP	Not performed	Negative	Not performed

*ANA* Anti-nuclear antibody (NR < 1:80), *Anti-CCP* Anti-cyclic citrullinated peptide, Anti-dsDNA (NR < 5 mg/L), Anti-smooth muscle (NR < 1:10), C Complement (< 5 mg/l), *CRP* C-reactive protein, *ENA* Extractable nuclear antigen (NR < 1:80), *Hb* Haemoglobin, *Ig* Immunoglobulin, *ILD* Interstitial lung disease, *NR* Normal range, *RF* Rheumatoid factor (NR < 30 IU/mL). ^a^On single measurement. ^b^First test was positive with value shown, second test was negative

Case 2 is the 22-year-old mother of case 1. She was diagnosed with RF^+^ polyarticular JIA in early childhood having initially presented with arthritis of her right knee which progressed to polyarticular involvement that included temporomandibular and hip joints. She was also noted to have a livedo-type rash on her legs. Results of her laboratory findings are shown in Table 1. She was treated with methotrexate, then mycophenolate and adalimumab with partial response. She achieved good disease control with rituximab at the age of 18 years, with no evidence of destructive arthropathy. Lung biopsy was performed at 8 years of age due to a persistent cough, and abnormal chest radiograph and pulmonary function tests (details not available). ILD was diagnosed, presumed secondary to methotrexate and JIA. ILD remained subclinical until 14 weeks of pregnancy when she developed increased work of breathing requiring admission and supplemental oxygen. A pneumocystis immunofluorescence test was negative, but she was treated for *pneumocystis jiroveci* pneumonia together with a tapering course of oral prednisolone. Post-partum her symptoms stabilised, and her ongoing supplemental oxygen requirement decreased.

Case 3 is the maternal grandmother of case 1. She was diagnosed with RF^+^ polyarticular JIA at 16 years of age which was initially treated with sulfasalazine, then sodium aurothiomalate. She subsequently developed breathing difficulties and a dry cough. Pulmonary function tests showed a restrictive defect (transfer factor of 32% predicted). Penicillamine was substituted for sodium aurothiomalate with subsequent respiratory improvement. Her symptoms recurred at 35 weeks in to her first pregnancy (Case 2). She was treated with oral prednisolone and had a normal delivery at 40 weeks. Respiratory symptoms resolved post-partum. ILD secondary to RA (rheumatoid arthritis) and associated treatment was the suggested diagnosis following lung biopsy. Her arthritis was subsequently treated with low dose methotrexate then azathioprine. Her respiratory function deteriorated again at 22 weeks gestation during a second pregnancy. Oral and intravenous steroid treatment was effective. A 3rd pregnancy was uneventful, throughout which she was treated with 10 to 20 mg oral prednisolone daily. Due to declining lung function she underwent single lung transplantation at the age of 30 years. She died aged 38 years from multi-organ failure presumed secondary to lung infection, having presented with acute respiratory failure and lower lobe opacification of her transplanted lung. Post-mortem identified the cause of death to be multiple organ dysfunction syndrome and fungal infection of the left lung.

**Fig. 1:**
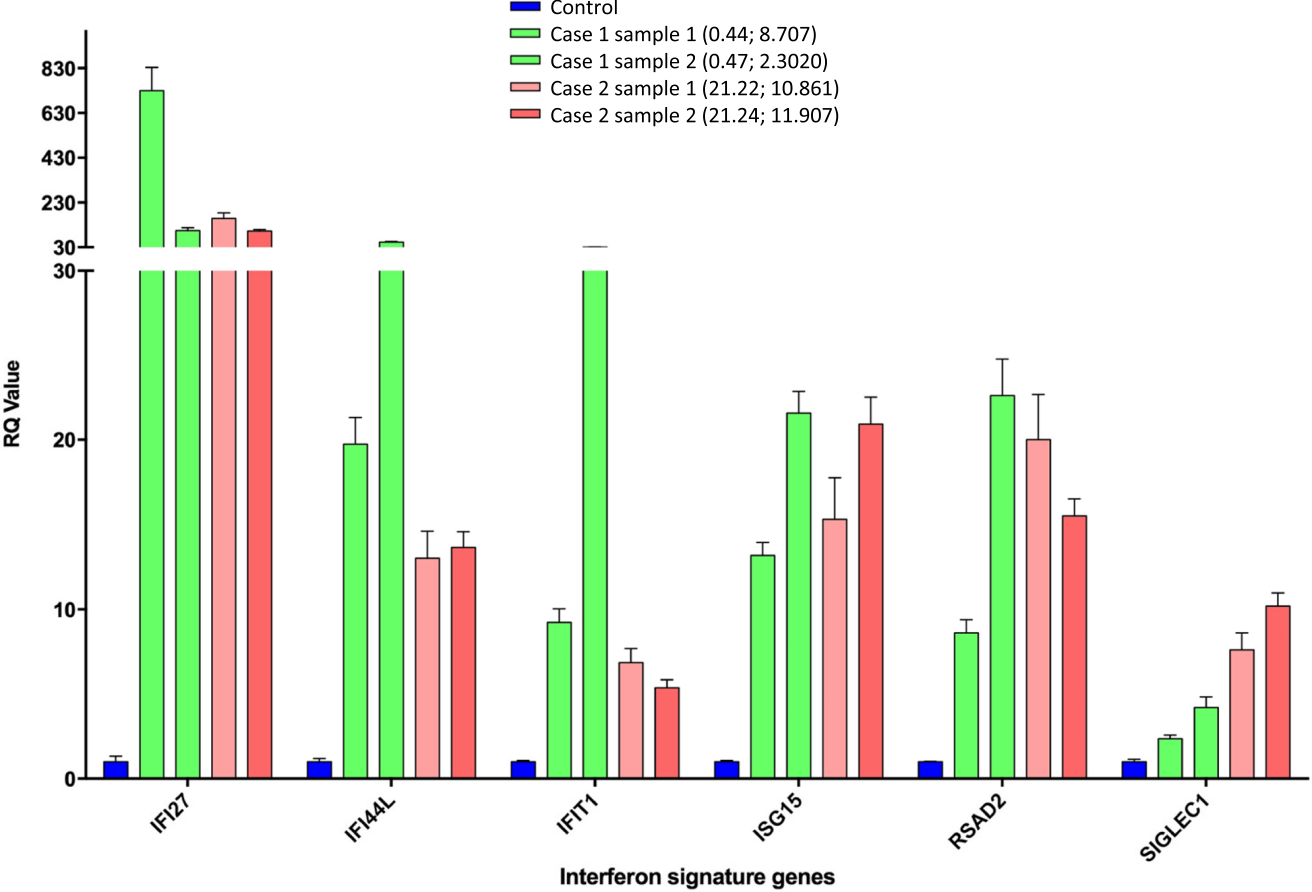
The expression of six interferon stimulated genes (ISGs) measured in whole blood from case 1 and case 2 on two different occasions was determined by quantitative reverse transcription PCR, and compared to the normalised data of 29 healthy controls (as described in Rice et al. Lancet Neurology 2013:12:1159–69). Numbers in brackets represent decimalized age (years) at time of sampling followed by the interferon score derived from the expression levels of interferon signature genes. RQ, relative quantification

The family history of ILD across three generations suggested an autosomal dominant aetiology, and in the context of JIA in cases 2 and 3, a diagnosis of coatomer protein subunit alpha (COPA) syndrome versus SAVI was proposed. Targeted Sanger sequencing of whole blood DNA from cases 1 and 2 was negative for mutations in *COPA* but identified a heterozygous *TMEM173* mutation (c.463G > A; p.Val155Met) in both patients, consistent with SAVI. DNA was subsequently extracted from stored appendicectomy tissue from case 3 and was shown to be positive for the same *TMEM173* mutation. RNA was extracted from peripheral whole blood from cases 1 and 2 to functionally assess the *TMEM173* mutation. The expression of six interferon stimulated genes was measured using quantitative PCR. An interferon score, calculated by comparing the median fold expression of these genes in case 1 and 2 against their expression in 29 healthy controls, was generated as previously described [5]. A prominent interferon signature was identified in both cases and confirmed on repeat testing (Fig. 1).

Following the molecular diagnosis, case 1 was treated with 3 days of 10 mg/kg intravenous methylprednisolone with limited clinical response followed by high oral prednisolone (which was later weaned). She also received 2 g/kg monthly intravenous immunoglobulin and was extubated onto high flow nasal cannula oxygen therapy following several failed extubation attempts. Due to the reported benefits of JAK1/2 inhibition in SAVI [6, 7], case 1 commenced treatment with baricitinib 1 mg twice daily at 8 months of age and weaned to low flow oxygen 1 month thereafter. She is currently stable from a respiratory perspective with mild tachypnoea and increased work of breathing. There is no evidence of joint disease.

Case 2 remained in remission with regards to her arthritis until 10 months post-partum, when she had a flare of arthritis for which she again received rituximab. She has since commenced baricitinib, initially at 2 mg daily then increased to 4 mg daily, which has been well tolerated. She remains on 1 L per minute low flow oxygen but reports an improvement in activities of daily living. Her oxygen requirement is anticipated to be long-term.

## Discussion

Since the term type 1 interferonopathy was first coined in 2011 [8], a growing number of genotypes and molecular pathways have been identified which, in the mutant state, lead to chronic upregulation of type 1 interferon [9]. SAVI was initially reported in 2014 as a pulmonary vascular syndrome with significant cutaneous involvement [1]. More recently it has been recognised that the associated phenotype is highly variable. The original description of SAVI is one of pulmonary, cutaneous and vascular manifestations. More recently it has been recognised that the associated phenotype is highly variable [10] with case descriptions ranging from lethal lung involvement to isolated cutaneous disease [1, 2, 4, 7, 11]. Similar to a recent SAVI case report of absent cutaneous involvement [12], none of the cases reported here had the typical, severe skin manifestations of the condition. Indeed, the variable phenotypic expression reported in familial cases [2–4] is well illustrated in our cases.

It is increasingly recognised that type 1 interferonopathies can manifest as musculoskeletal disease. In 2017, Carvalho et al reported two families demonstrating an autosomal dominant, multisystem syndrome with progressive musculoskeletal disease which presented similarly to Jaccoud’s arthropathy [13]. Affected cases carried a heterozygous gain-of-function mutation in *IFIH1* (the gene encoding MDA5), resulting in chronic upregulation of type 1 interferon signalling. More recently, Rodero et al described a case of deforming arthropathy in the context of biallelic hypomorphic mutations in *DNASE2*, leading to loss of DNase II activity, an accumulation of self-derived DNA and subsequent type 1 interferon production [14]. Arthritis (including RF^+^ arthritis), arthralgia and myalgia have previously been described in the SAVI literature. However, when present, these features have generally been reported as minor features of the condition [1–3, 7]. To our knowledge this family is apparently so-far unique, whereby SAVI presented as a non-destructive, non-deforming RF^+^ polyarticular JIA in the two older cases. It remains to be seen whether the proband will also manifest arthritis while receiving a JAK1/2 inhibitor.

## Conclusion

ILD is commonly associated with connective tissue disorders and their treatments [15]. However, ILD presenting in three generations of the same family in the context of RF^+^ polyarticular JIA triggered investigation for an underlying autosomal dominant cause. A unifying diagnosis of SAVI has allowed more targeted therapy and significantly altered disease counselling for this family. Thus, this report highlights the need for physicians to revisit the diagnoses of patients with complex, atypical or treatment-resistant disease. We urge rheumatology and respiratory physicians to consider a type 1 interferonopathy in patients with JIA/early onset RA plus ILD.

## Data Availability

Not applicable.

## Abbreviations

ANA: Antinuclear antibody
C: Complement
CCP: Cyclic citrullinated peptide
CRP: C-reactive protein
ENA: Extractable nuclear antigen
Hb: Haemoglobin
Ig: Immunoglobulin
ILD: Interstitial lung disease
JIA: Juvenile rheumatoid arthritis
NR: Normal range
RA: Rheumatoid arthritis
RF: Rheumatoid factor
SAVI: STING-associated vasculopathy with onset in infancy
STING: Stimulator of interferon genes

## References

[1] Liu Y (2014). Activated STING in a vascular and pulmonary syndrome. N Engl J Med.

[2] Jeremiah N (2014). Inherited STING-activating mutation underlies a familial inflammatory syndrome with lupus-like manifestations. J Clin Invest.

[3] Picard C (2016). Severe pulmonary fibrosis as the first manifestation of Interferonopathy (TMEM173 mutation). Chest.

[4] König N (2017). Familial chilblain lupus due to a gain-of-function mutation in STING. Ann Rheum Dis.

[5] Rice GI (2013). Assessment of interferon-related biomarkers in Aicardi-Goutières syndrome associated with mutations in TREX1, RNASEH2A, RNASEH2B, RNASEH2C, SAMHD1, and ADAR: a case-control study. Lancet Neurol.

[6] Sanchez GAM (2018). JAK1/2 inhibition with baricitinib in the treatment of autoinflammatory interferonopathies. J Clin Invest.

[7] Frémond ML (2016). Efficacy of the Janus kinase 1/2 inhibitor ruxolitinib in the treatment of vasculopathy associated with TMEM173-activating mutations in 3 children. J Allergy Clin Immunol.

[8] Crow YJ (2011). Type I interferonopathies: a novel set of inborn errors of immunity. Ann N Y Acad Sci.

[9] Rodero MP, Crow YJ (2016). Type I interferon–mediated monogenic autoinflammation: the type I interferonopathies, a conceptual overview. J Exp Med.

[10] Clarke SL (2016). Interstitial lung disease caused by STING-associated vasculopathy with onset in infancy. Am J Respir Crit Care Med.

[11] Omoyinmi E (2015). Stimulator of interferon genes-associated vasculitis of infancy. Arthritis Rheum.

[12] Raffaele CGL, et al. A patient with stimulator of interferon genes–associated vasculopathy with onset in infancy without skin vasculopathy. Rheumatology. 2019;59:905–7. 10.1093/rheumatology/kez444 31598716

[13] de Carvalho LM (2017). Musculoskeletal disease in MDA5-related type I Interferonopathy: a Mendelian mimic of Jaccoud's Arthropathy. Arthritis Rheum.

[14] Rodero MP (2017). Type I interferon-mediated autoinflammation due to DNase II deficiency. Nat Commun.

[15] Picchianti Diamanti A, Germano V, Bizzi E, Laganà B, Migliore A (2011). Interstitial lung disease in rheumatoid arthritis in the era of biologics. Pulmonary Med.

